# Supplementary figures and images for: A highly effective therapeutic ointment for treating corals with black band disease
Source: PLoS One. 2022 Oct 26;17(10):e0276902. doi: 10.1371/journal.pone.0276902 (PMC9605335; doi:10.1371/journal.pone.0276902)

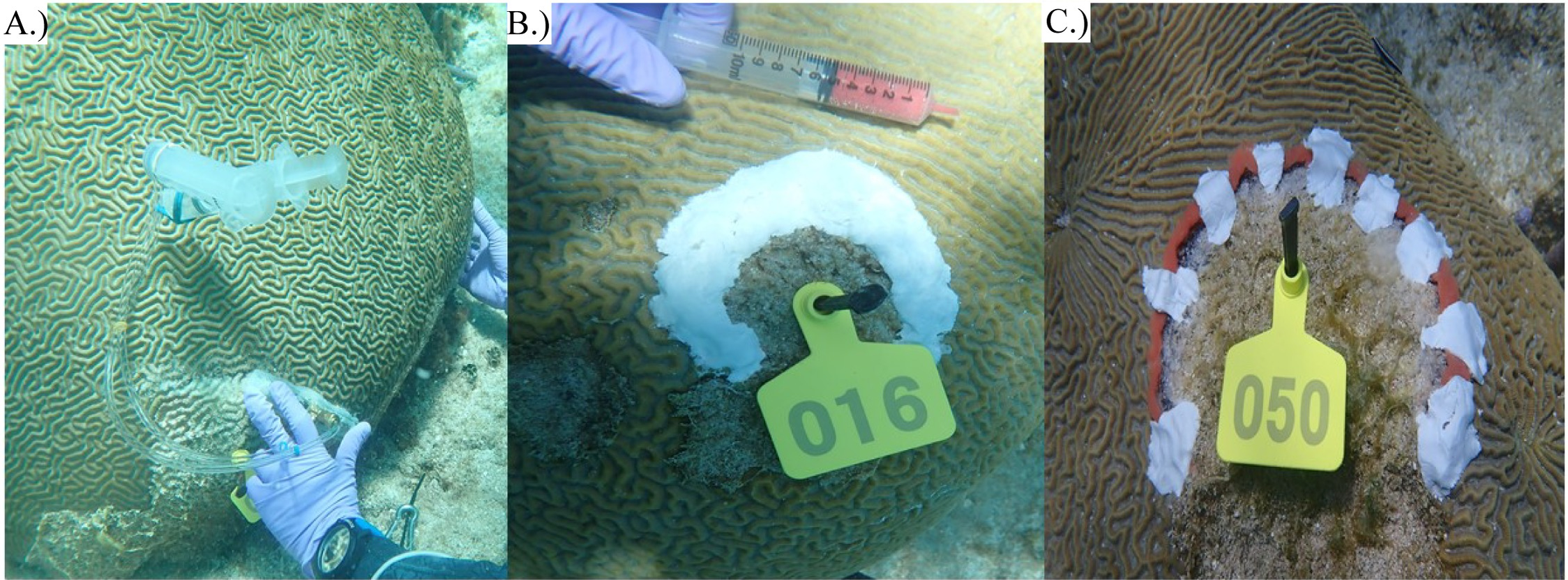

Supplement: S1 Fig — (A) H2O2 prototype being held over the black band disease (BBD) lesion, (B) Tooth whitening gel (not visible) applied to a BBD lesion and held in place with marine epoxy (white), (C) Tooth whitening gel (red ointment) mixed with Base2B applied to a BBD lesion, and anchored in place with marine epoxy (white). (TIF) [file pone.0276902.s001.tif]

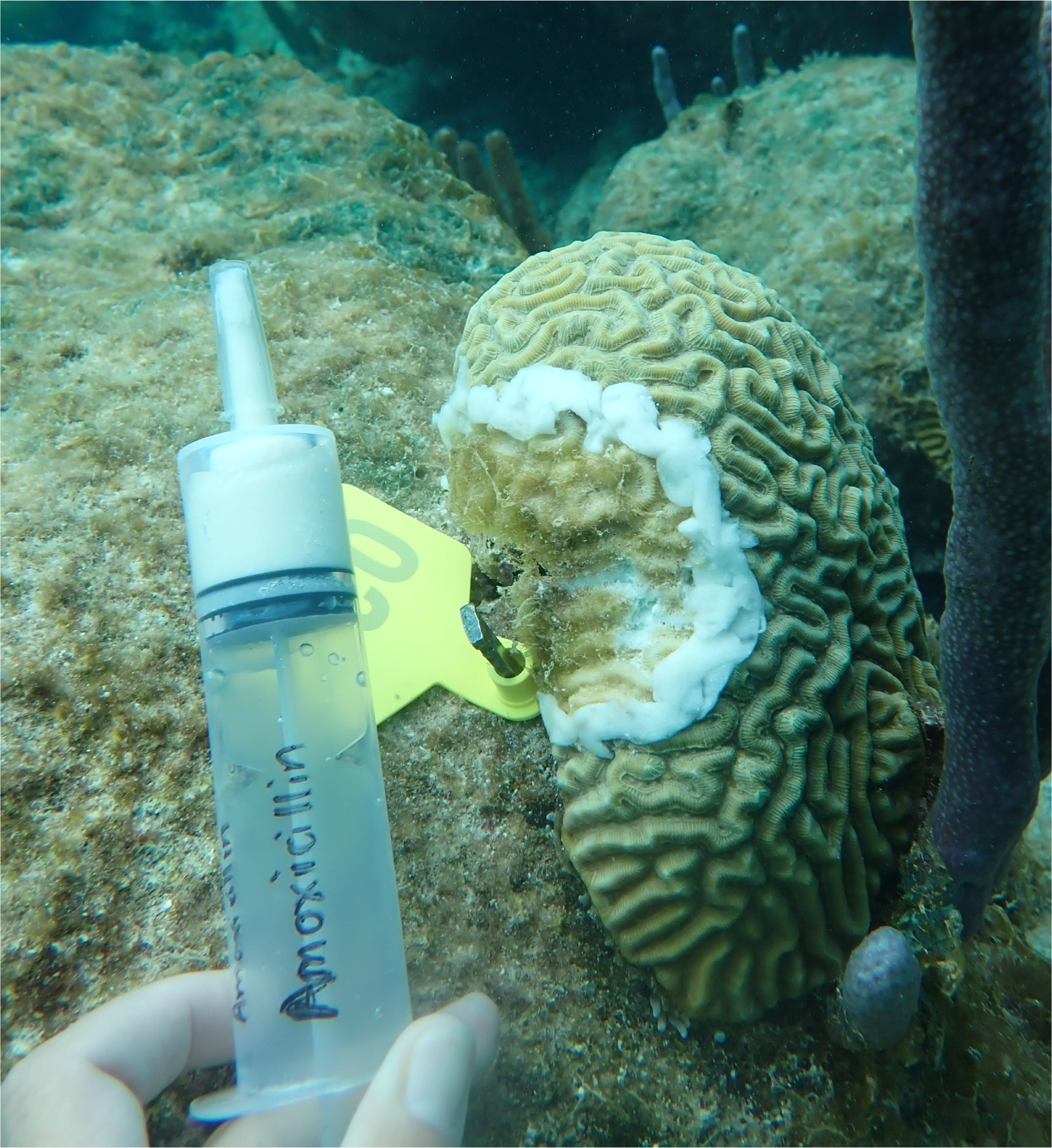

Supplement: S2 Fig — (TIF) [file pone.0276902.s002.tif]

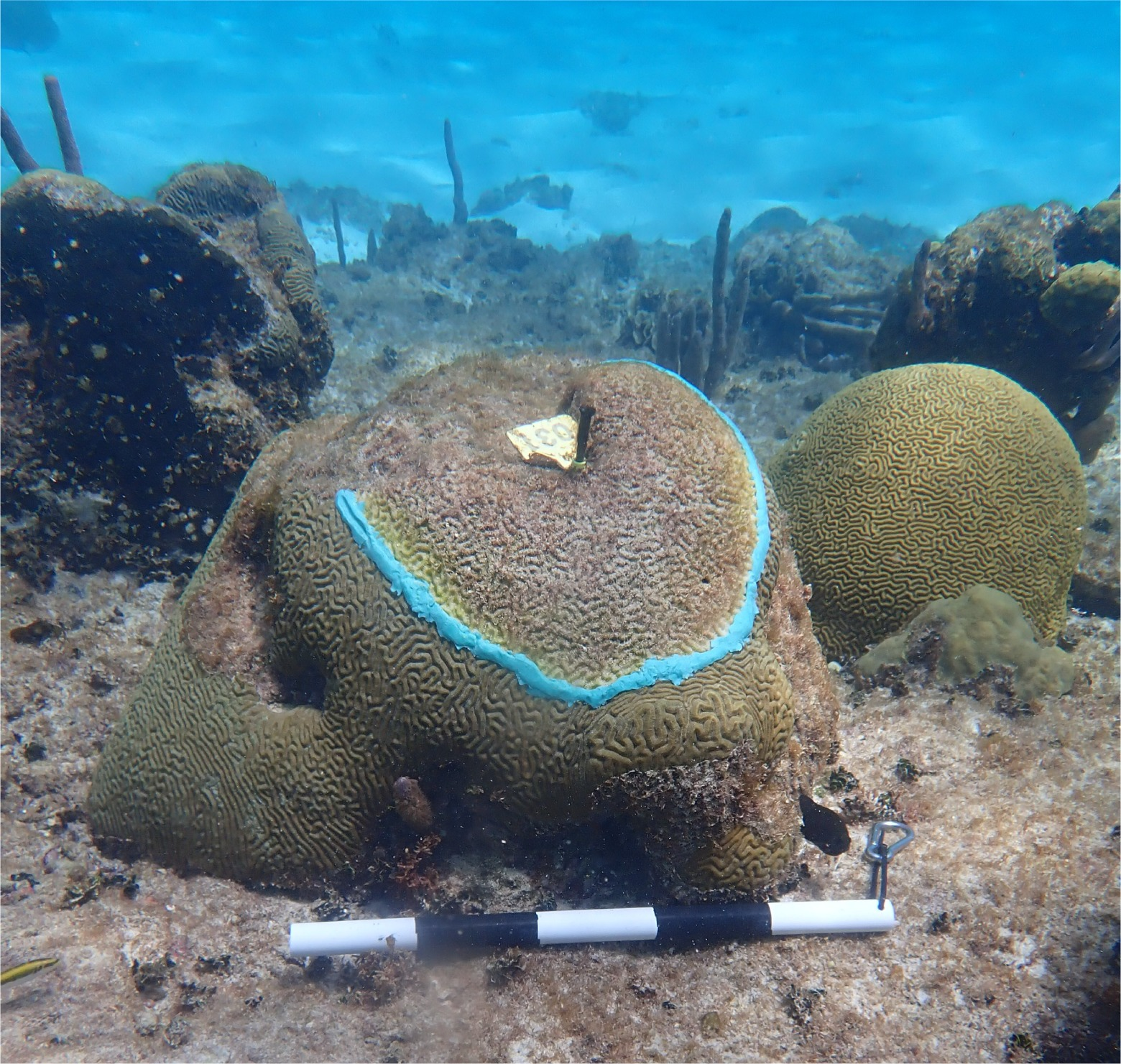

Supplement: S3 Fig — (TIF) [file pone.0276902.s003.tif]

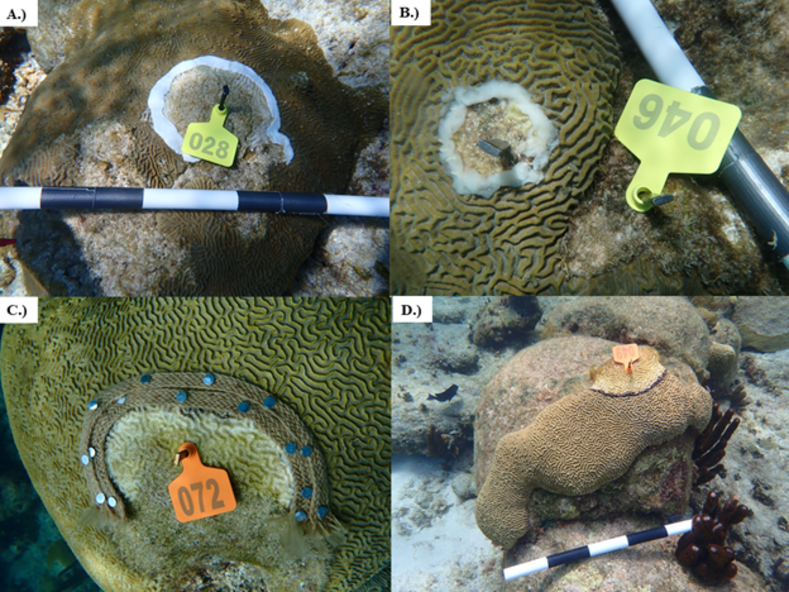

Supplement: S4 Fig — (A) Marine epoxy covering the BBD lesion, (B) Base2B applied to a black band disease (BBD) lesion, (C) Two pieces of jute rope covering the BBD lesion and apparently healthy tissue adjacent to the lesion, (D) Untreated control lesion. (TIF) [file pone.0276902.s004.tif]

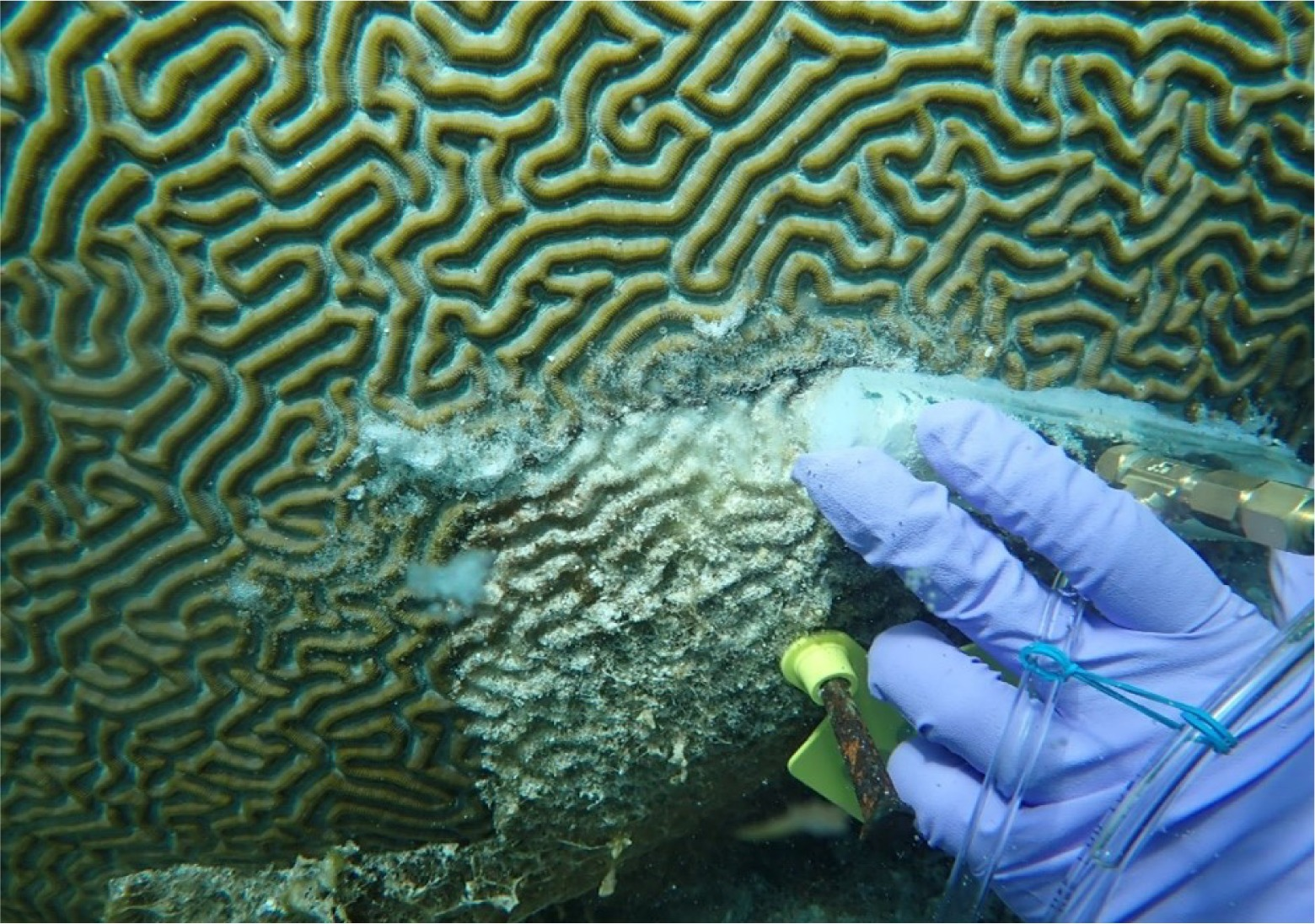

Supplement: S5 Fig — (TIF) [file pone.0276902.s005.tif]

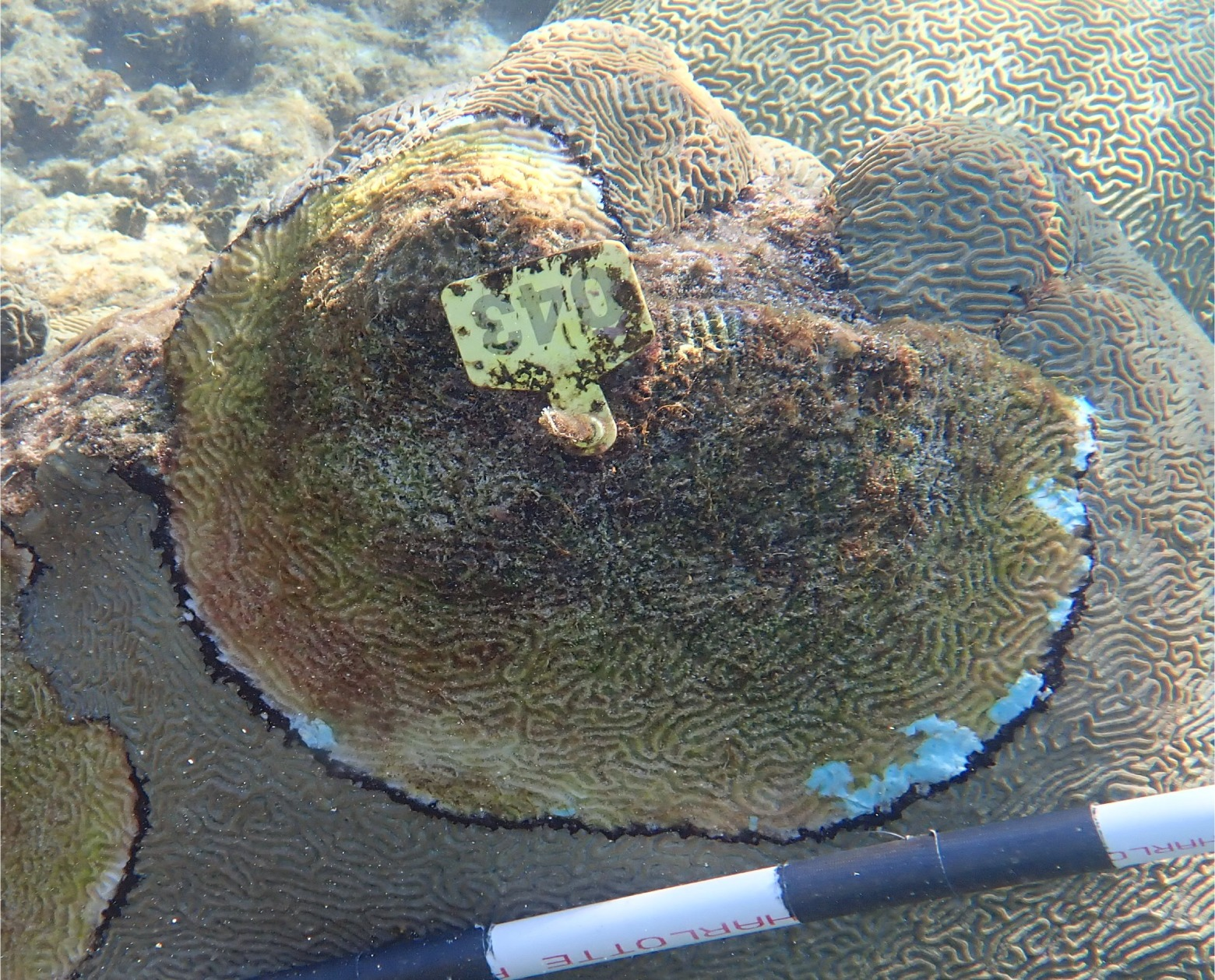

Supplement: S6 Fig — (TIF) [file pone.0276902.s006.tif]

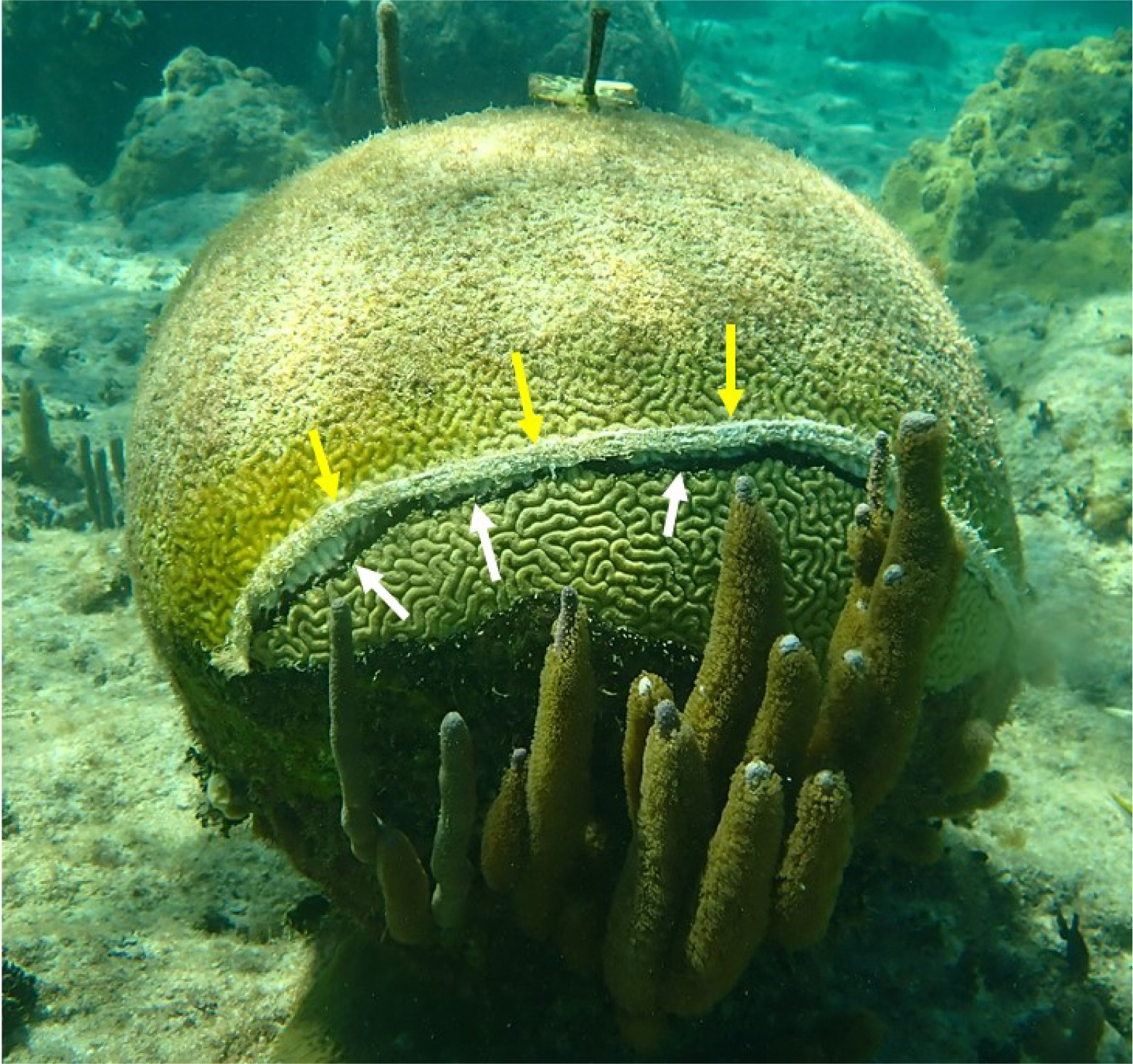

Supplement: S7 Fig — Yellow arrows represent where the BBD lesion started; white arrows indicate where the lesion migrated beyond the rope. (TIF) [file pone.0276902.s007.tif]
