# Supplementary material for: A highly effective therapeutic ointment for treating corals with black band disease
Source: PLoS One. 2022 Oct 26;17(10):e0276902. doi: 10.1371/journal.pone.0276902 (PMC9605335; doi:10.1371/journal.pone.0276902)
Supplement: S1 Table — Treatments were applied to Pseudodiploria spp. colonies in July 2020 and October 2020. (DOCX) [file pone.0276902.s008.docx]

| **Date of treatment** | **Treatment comparison** | **Statistical values** |
| --- | --- | --- |
| July 2020 | H_2_O_2_ prototype vs. control | F_1,8_ = 0.4236,  p = 0.5334 |
| July 2020 | Dental gel vs. control | F_1,8_ = 1.5412,  p = 0.2496 |
| July 2020 | Dental gel vs. epoxy only | H(1)=0.2727, p=0.6015 |
| July 2020 | Dental gel vs. Base2B only | H(1)=1.32, p=0.2506 |
| July 2020 | Dental gel + Base2B vs. control | F_1,8_ = 0.6236,  p = 0.4525 |
| July 2020 | Dental gel + Base2B vs. Base2B only | F_1,8_ = 0.0196,  p = 0.8921 |
| July 2020 | Amoxicillin + Base2B vs. control | H(1)=1.8436, p=0.1745 |
| July 2020 | Amoxicillin + Base2B vs. Base2B only | H(1)=0.8836, p=0.3472 |
| October 2020 | Amoxicillin + Base2B vs. control | F_1,8_ = 2.3069,  p = 0.1673 |
| October 2020 | CoralCure A ointment vs. control | H(1)=0.8333, p=0.3613 |
| October 2020 | CoralCure A rope vs. control | H(1)=0.0981, p=0.754 |
| October 2020 | CoralCure B ointment vs. control | F_1,9_ = 0.7488,  p = 0.4093 |
| October 2020 | CoralCure B rope vs. control | F_1,8_ = 0.1799,  p = 0.6826 |
| October 2020 | CoralCure C ointment vs. control | F_1,8_ = 0.0516,  p = 0.8259 |
| October 2020 | CoralCure C rope vs. control | H(1)=1.8894, p=0.1693 |
