# Supplementary material for: A highly effective therapeutic ointment for treating corals with black band disease
Source: PLoS One. 2022 Oct 26;17(10):e0276902. doi: 10.1371/journal.pone.0276902 (PMC9605335; doi:10.1371/journal.pone.0276902)
Supplement: S2 Table — (DOCX) [file pone.0276902.s009.docx]

| **Date of treatment** | **Treatment comparison** | **No. of treated corals (n=5) with active BBD lesions at revisit** | **No. of control corals (n=5) with active BBD lesions at revisit** | **Statistical values (Fisher’s Exact)** |
| --- | --- | --- | --- | --- |
| July 2020 | H_2_O_2_ prototype vs. control | 3 | 3 | p = 1 |
| July 2020 | Dental gel vs. control | 2 | 3 | p = 1 |
| July 2020 | Dental gel vs. epoxy only | 2 | 2 | p = 1 |
| July 2020 | Dental gel vs. Base2B only | 2 | 2* | p = 1 |
| July 2020 | Dental gel + Base2B vs. control | 2 | 3 | p = 1 |
| July 2020 | Dental gel + Base2B vs. Base2B only | 2 | 2* | p = 1 |
| July 2020 | Amoxicillin + Base2B vs. control | 2 | 3 | p = 1 |
| July 2020 | Amoxicillin + Base2B vs. Base2B only | 2 | 2* | p = 1 |
| October 2020 | Amoxicillin + Base2B vs. control | 1 | 1 | p = 1 |
| October 2020 | CoralCure A ointment vs. control | 3 | 1 | p = 0.524 |
| October 2020 | CoralCure A rope vs. control | 0 | 1 | p = 1 |
| October 2020 | CoralCure B ointment vs. control | 1 | 1 | p = 1 |
| October 2020 | CoralCure B rope vs. control | 3 | 1 | p = 0.524 |
| October 2020 | CoralCure C ointment vs. control | 0 | 1 | p = 1 |
| October 2020 | CoralCure C rope vs. control | 1* | 1 | p = 1 |

***= coral was dead at time of revisit**
