# Supplementary material for: A highly effective therapeutic ointment for treating corals with black band disease
Source: PLoS One. 2022 Oct 26;17(10):e0276902. doi: 10.1371/journal.pone.0276902 (PMC9605335; doi:10.1371/journal.pone.0276902)
Supplement: Attachment — Submitted filename: Response to reviewers (2).docx [file pone.0276902.s010.docx]

October 10, 2022

Dear Members of the Editorial Board,

Please consider the revised manuscript titled “A highly effective therapeutic ointment for treating corals with black band disease” for publication as a research article in PLoS ONE. On 10/6/2022 the journal requested an additional revision before publishing. This manuscript was revised to address this point.

Within the manuscript, “Table 7” should be called “Table 5”. This was changed in the manuscript in lines 343 and 345. We thank the academic editor and reviewers for providing thoughtful comments and suggestions, which have helped improve the clarity of the manuscript. All the figures presented in this manuscript are my own and I have no interests, financial or personal, to declare. Please let me know if any additional information is needed to publish the manuscript.

Sincerely,

Katherine R. Eaton

Corresponding author
